# 17β-Estradiol Enhances Breast Cancer Cell Motility and Invasion via Extra-Nuclear Activation of Actin-Binding Protein Ezrin

**DOI:** 10.1371/journal.pone.0022439

**Published:** 2011-07-26

**Authors:** Shuhui Zheng, Jinghe Huang, Kewen Zhou, Chengxi Zhang, Qiuling Xiang, Zhi Tan, Tinghuai Wang, Xiaodong Fu

**Affiliations:** 1 Department of Physiology, Zhongshan School of Medicine, Sun Yat-sen University, Guangzhou, China; 2 Department of Cardiovascular Internal Medicine, The Third Affiliated Hospital, Sun Yat-sen University, Guangzhou, China; Institut de Génomique Fonctionnelle de Lyon, France

## Abstract

Estrogen promotes breast cancer metastasis. However, the detailed mechanism remains largely unknown. The actin binding protein ezrin is a key component in tumor metastasis and its over-expression is positively correlated to the poor outcome of breast cancer. In this study, we investigate the effects of 17β-estradiol (E2) on the activation of ezrin and its role in estrogen-dependent breast cancer cell movement. In T47-D breast cancer cells, E2 rapidly enhances ezrin phosphorylation at Thr^567^ in a time- and concentration-dependent manner. The signalling cascade implicated in this action involves estrogen receptor (ER) interaction with the non-receptor tyrosine kinase c-Src, which activates the phosphatidylinositol-3 kinase/Akt pathway and the small GTPase RhoA/Rho-associated kinase (ROCK-2) complex. E2 enhances the horizontal cell migration and invasion of T47-D breast cancer cells in three-dimensional matrices, which is reversed by transfection of cells with specific ezrin siRNAs. In conclusion, E2 promotes breast cancer cell movement and invasion by the activation of ezrin. These results provide novel insights into the effects of estrogen on breast cancer progression and highlight potential targets to treat endocrine-sensitive breast cancers.

## Introduction

Breast cancer is the most frequently diagnosed cancer in women. Despite the recent improvements in survival rates, many patients relapse and die for disseminated metastatic disease, which supports the demand for new therapeutic strategies.

Female sex steroid estrogen is the fundamental regulator of normal breast development. On the other side, the risk of breast cancer is associated with early menarche, late menopause and hormone replacement therapy (HRT) [1]. All these factors increase the duration of exposure of breast tissue to estrogen, which facilitates breast cancer initiation through multiple established mechanisms [2]. In addition to this, clinical data showed that the adjuvant therapy with aromatase inhibitors reduces early distant metastasis and improves disease-free survival [3], indicating that estrogen may facilitate the progression of breast cancer. However, the mechanistic basis of estrogen on breast tumor cell motility or invasion remains unclear.

Cell migration is required for cancer spread, invasion, and metastasis. The primary mechanism for most types of cell migration is the actin cytoskeleton remodeling [4]. In this process, G-actin readily polymerizes to form F-actin and *de novo* actin polymerization occurs at the leading edge, resulting in the formation of membrane protrusions such as filopodia, lamellipodia as well as invadopodia. The actin-binding ERM (ezrin/radixin/moesin) proteins, concentrated in actin rich cell-surface structures, are important regulators of actin reorganization by cross-linking actin filaments with the plasma membrane [5]. We recently discovered that sex steroids, including estrogen and progesterone, promote endothelial as well as breast cancer cell migration by inducing the formation of membrane protrusions [6], [7], [8], [9]. These events are dependent on the rapid activation of moesin, one member of ERM family. However, moesin is found primarily in endothelial cells whereas ezrin is concentrated in epithelial cells [10], [11]. Therefore, the role of ezrin in sex steroids-sensitive breast cancer metastasis should not be neglected.

Clinical data have indicated the positive correlation between ezrin expression/abnormal localization and advancing histological grade/poor outcome [12], [13]. Additionally, animal model experiment demonstrated that the overexpression of wild-type ezrin promotes breast cancer metastasis while expression of the dominant-negative amino-terminal ezrin domain markedly inhibits metastasis [14]. In quiescent conditions ezrin exists in an auto-inhibited conformation and phosphorylation on the carboxyl-terminal threonine 567 (Thr^567^) leads to ezrin activation, which subsequently associates membrane associated adhesion molecules on ezrin's NH2-terminal end and with polymerized F-actin on ezrin's COOH-terminal, leading to actin reorganization and the formation of membrane structures [15]. Several protein kinases, including protein kinase C, G protein-coupled receptor kinase 2 and Rho kinase have been proposed to mediate the threonine phosphorylation of ezrin in vitro and/or in vivo [16], [17], [18]. However, whether ezrin activity is modulated by sex steroids is still a matter of investigation.

In this manuscript, we explored the regulatory actions of estrogen on ezrin activity and the effects on breast cancer cell cytoskeleton remodeling, migration and invasion. Moreover, we characterized the signaling pathways initiated by estrogen receptor that lead to ezrin activation.

## Results

### E2 triggers a rapid phosphorylation of the actin-binding protein ezrin

**Figure 1:**
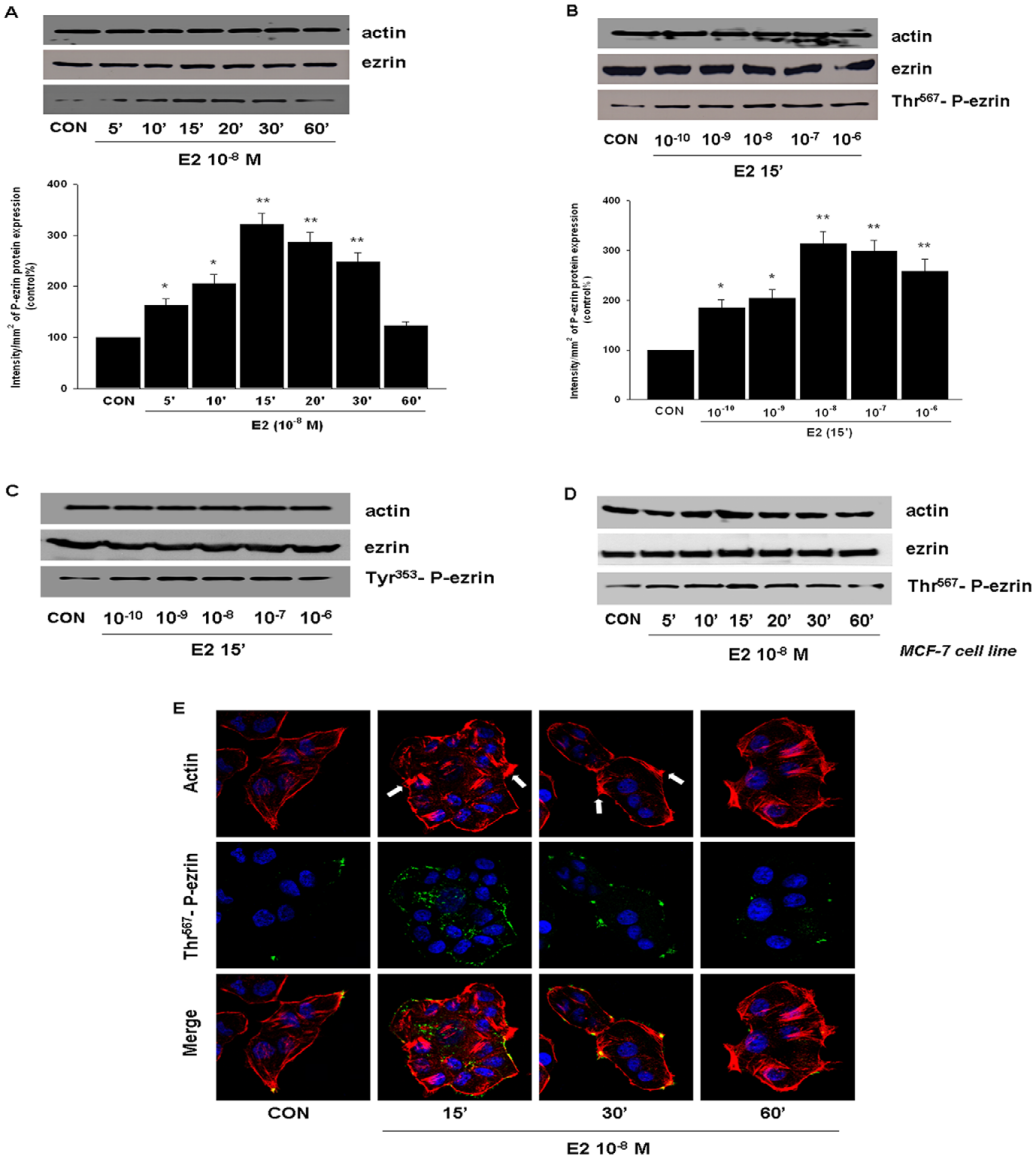
E2 activates ezrin and induces rapid actin cytoskeleton rearrangement in T47-D cells. (A) and (B) show the time- and dose-dependent ezrin activation in T47-D breast cancer cells after treatment with E2. Total cell amount of wild-type (ezrin) or Thr^567^-phosphorylated ezrin (P-ezrin) are shown with western blot. P-ezrin densitometry values were adjusted to ezrin intensity, then normalized to expression from the control sample. * = P<0.05 vs. corresponding control; ** = P<0.01 vs. corresponding control. (C) shows dose-dependent ezrin phosphorylation at Tyr^353^ after treatment with E2 for 15 minutes in T47-D cells. Tyr^353^-phosphorylated ezrin (P-ezrin) are shown with western blot and actin is taken as the loading control. (D) In MCF-7 breast cancer cells, E2 induced ezrin phosphorylation at Thr^567^ in a time-dependent manner. (E) T47-D cells were treated with E2 (10^−8^ M) for the indicated time. Then the cells were stained with anti-phospho-Thr^567^ ezrin (P-ezrin) linked to FITC. Actin was stained with phalloidin linked to Texas Red and nuclei were counterstained with DAPI. White arrows indicate the lamellipodia. All the experiments were repeated three times with consistent results, and the representative images are shown.

Treatment with E2 (10^−8^ M) on T47-D breast cancer cells resulted in a rapid increase of Thr^567^-phosphorylation of ezrin (Fig. 1A). This phenomenon was time dependent and transient, being maximal after 10–15 min and reversing to baseline after 60 min (Fig. 1A). Total ezrin did not change during this time frame (Fig. 1A). Meanwhile, ezrin phosphorylation was found throughout a range of E2 concentrations that fall within the physiological range (Fig. 1B).

Thr^567^-phosphorylation corresponds to ezrin activation [15] and Tyr^353^ phosphorylation is required for its full activation [19]. Indeed, when cells were exposed to different concentrations of E2 (10^−10^–10^−6^ M) for 15 min, Tyr^353^ phosphorylation of ezrin was significantly enhanced (Fig. 1C).

Likewise, in ER/PR positive MCF-7 breast cancer cells, E2 also provoked the rapid phosphorylation of ezrin on Thr^567^, suggesting that this action is generalized rather than cell specific (Fig. 1D).

Actin fibers in T47-D breast cancer at baseline were arranged longitudinally in the cytoplasm and the cell membrane was regular. Treatment with E2 (10^−8^ M) resulted in a rapid shift of the actin fibers toward the edge of the membrane (Fig. 1E). In parallel, cell membrane specialized structures, such as lamillipodia, were formed at sites enriched in actin where it colocalized with phosphorylated ezrin (Fig. 1E). These effects were visible in 15 minutes and began to revert after 30 minutes (Fig. 1E).

### ERα supports the extra-nuclear signaling of E2 to ezrin

**Figure 2:**
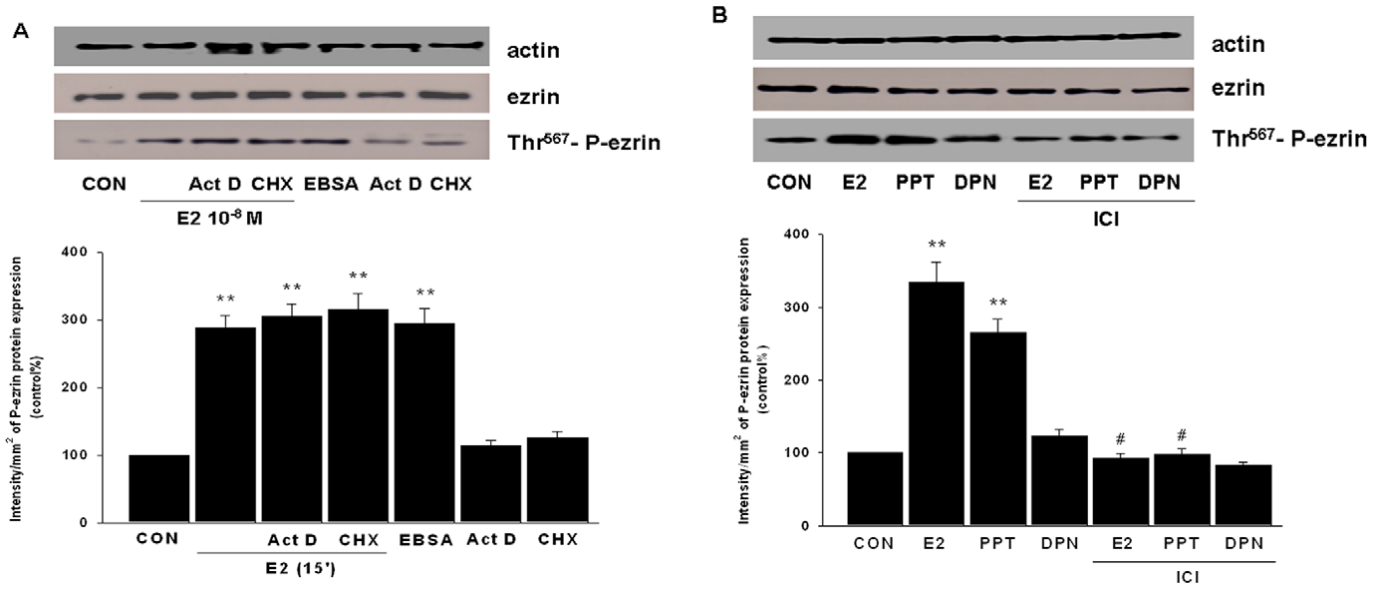
ERα supports the extra-nuclear signaling of E2 to ezrin. (A) T47-D cells were treated with E2 (10^−8^ M) or E-BSA (10^−8^ M) for 15 min, in the presence or absence of actinomycin D (Act D - 10 µM) or cycloheximide (CHX - 200 µM). Ezrin and phosphorylated ezrin are shown. ** = P<0.01 vs control. (B) T47-D cells were treated with E2 (10^−8^ M), PPT (10^−9^ M) or DPN (10^−9^ M) for 15 min, in the presence or absence of ICI 182,780 (ICI - 1 µM). Ezrin and phosphorylated ezrin are shown. ** = P<0.01 vs. control. # = P<0.01 vs. E2 or PPT, respectively. All the experiments were repeated three times with consistent results, and the representative images are shown.

The rapid time-lapse of ezrin activation and de-activation suggests that it is likely to be mediated by extra-nuclear pathways [20]. Indeed, activation of estrogen receptor (ER) with E2 still resulted in ezrin activation even if RNA or protein synthesis was blocked with actinomycin D (Act D - 10 mM) or cycloheximide (CHX - 200 mM) (Fig. 2A). Likewise, estradiol-bovine serum albumin conjugate (EBSA, 10^−8^ M), a membrane -impermeable form of E2, also led to rapid activation of ezrin (Fig. 2A).

To identify which ER isoform is required for the signaling of E2 to ezrin, T47-D cells were treated with E2 (10^−8^ M), with the preferential ERα agonist PPT (10^−9^ M) or with the ERβ agonist DPN (10^−9^ M). As shown in Fig. 2B, ezrin activation was triggered only by E2 or PPT, which was largely prevented by the addition of the pure ER antagonist ICI 182,780 (ICI - 1 µM). In contrast, DPN failed to activate ezrin (Fig. 2B). These findings indicate that ERα supports the signaling of E2 to ezrin while ERβ is not required.

### ERα signaling to ezrin requires c-Src and phosphatidylinositol-3-kinase (PI3K)/Akt

**Figure 3:**
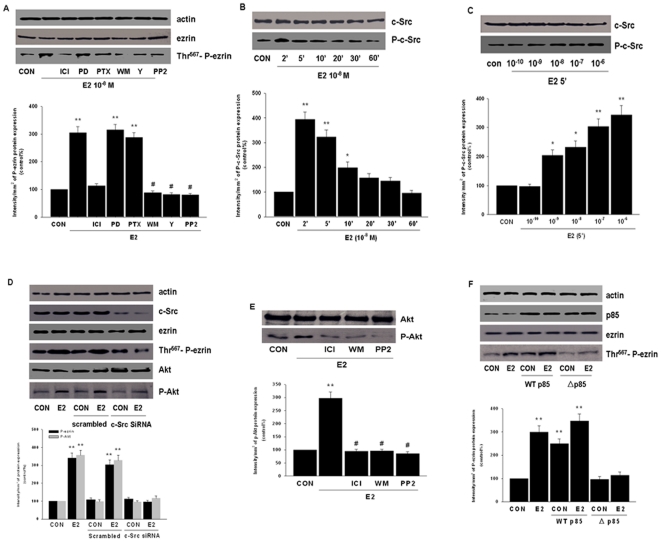
c-Src and phosphatidylinositol-3-kinase (PI3K)/Akt are implicated in E2-induced ezrin phosphorylation. (A) T47-D cells were exposed to 10^−8^ M E2 for 15 min, in the presence or absence of the pure ER antagonist ICI 182,780 (ICI - 1 µM), of the MEK inhibitor PD98059 (PD - 5 µM), of the G protein inhibitor, PTX (100 ng/mL), of the PI3K inhibitor wortmannin (WM - 30 nM), of the ROCK-2 inhibitor, Y-27632 (Y - 10 µM) or of the c-Src kinase inhibitor, PP2 (10 µM). Cell content of wild-type or phosphorylated ezrin are shown. P-ezrin densitometry values were adjusted to ezrin intensity and then normalized to expression from the control sample. ** = P<0.01 vs. control, # = P<0.01 vs. E2. (B) and (C) show the time- and dose-dependent c-Src activation in T47-D breast cancer cells after treatment with E2. Total cell amount of wild-type (c-Src) or Tyr^416^-phosphorylated c-Src (P-c-Src) are shown with western blot. P-c-Src densitometry values were adjusted to c-Src intensity, then normalized to expression from the control sample. (D) T47-D cells were exposed to 10^−8^ M E2 for 15 min after transfection with 100 nM c-Src siRNAs or control scrambled siRNAs for 48 h. Total actin, c-Src, ezrin, P-ezrin, Akt and P-Akt amounts and statistics for densitometry are shown. ** = P<0.01 vs. corresponding control. (E) T47-D cells were treated with 10^−8^ M E2 for 15 min, with or without the ER antagonist ICI 182,780 (ICI - 1 µM), PI3K inhibitor wortmannin (WM - 30 nM) or c-Src kinase inhibitor, PP2 (10 µM). Active Akt (P-Akt) densitometry values were adjusted to wild-type Akt intensity, then normalized to expression from the control sample. ** = P<0.01 vs. control. # = P<0.01 vs. P. (F) Cells were exposed to 10 nM E2 for 15 min after transfection with wild type p85α (WT p85α, 1.5 µg) or dominant-negative p85α (Δp85α, 1.5 µg) for 48 h. Cell contents of actin, p85 protein, wild-type and phosphorylated ezrin and statistics for densitometry are shown. ** = P<0.01 vs. corresponding control without transfection. All these experiments were performed in triplicates and representative images are shown.

To clarify the implicated signaling intermediates, some of the cascades responsible for extra-nuclear actions were interfered by using signaling inhibitors. Consistently, E2-induced ezrin activation was prevented by ICI 182,780 (ICI - 1 µM) (Fig. 3A). On the opposite, ezrin phosphorylation was not altered by the addition of the mitogen-activated protein kinase kinase (MEK) inhibitor PD98059 (PD - 5 µM) or of the G protein inhibitor pertussis toxin (PTX - 100 ng/mL) (Fig. 3A). However, PI3K inhibitor wortmannin (WM - 30 nM), Rho-associated kinase 2 (ROCK-2) Y27632 (Y - 10 µM) and non-receptor tyrosine kinase c-Src inhibitor PP2 (10 µM) all largely impaired E2-induced ezrin activation (Fig. 3A), indicating that c-Src, PI3K and ROCK-2 are required for this action.

To confirm the role of c-Src, the level of phospho- Tyr^416^-Src, which corresponds to its activation [21], was observed. Treatment with E2 (10^−8^ M) led to an increase of phospho- Tyr^416^-Src at 2 to 10 minutes and then it declined after 20 minutes (Fig. 3B). In addition, c-Src phosphorylation was increased when cells were treated with a range of E2 concentrations for 5 minutes (10^−10^–10^−6^ M) (Fig. 3C). When c-Src expression was silenced with specific siRNAs, E2 (10^−8^ M) failed to activate ezrin (Fig. 3D).

ERα interacts with the non-receptor tyrosine kinase c-Src and this action links to the activation of PI3K [22]. This was confirmed by our results showing that E2 (10^−8^ M) induced an increase of phosphorylation of the PI3K downstream effector, protein kinase Akt when compared to the vehicle-treated control (Fig. 3E). The transfection with c-Src siRNA or the use of c-Src inhibitor PP2 both blocked E2-induced Akt phosphorylation (Fig. 3D, 3E), suggesting that c-Src is the upstream of PI3K/Akt. In addition, transfection of T47-D cells with a dominant negative form of the regulatory subunit of PI3K, p85α (Δp85α), resulted in the impairment of the E2-dependent ezrin activation (Fig. 3F). As control, the transfection of a wild-type p85α construct (WT p85α) did not alter ezrin activation (Fig. 3F). The p85 plasmids were overexpressed in both conditions, implying an efficient transfection in our experimental settings (Fig. 3F).Taken together; these results suggest that the c-Src-dependent activation of PI3K/Akt is implicated in E2-induced ezrin activation.

### ER signaling to ezrin: role of RhoA and ROCK-2

**Figure 4:**
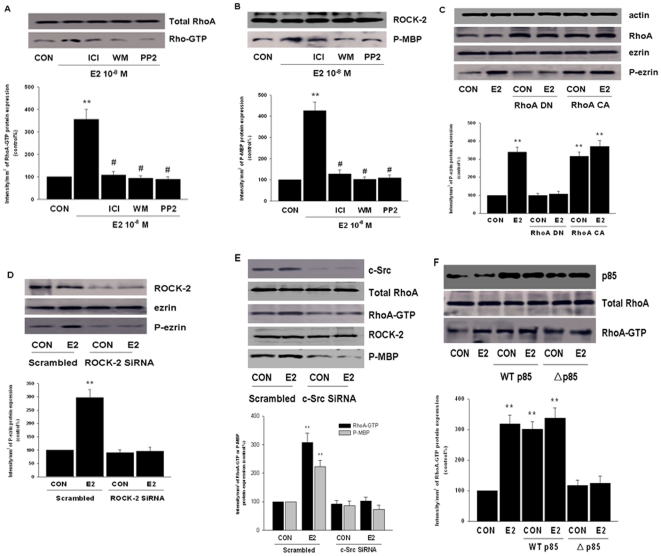
RhoA and ROCK-2 are activated during ER signaling to ezrin. (A) RhoA activity was assayed in cells treated with E2 (10^−8^ M) for 15 min in the presence or absence of the ICI 182,780 (ICI - 1 µM), of the PI3K inhibitor wortmannin (WM - 30 nM), or of the c-Src kinase inhibitor, PP2 (10 µM). Active, GTP-bound RhoA was immunoprecipitated with Rhotekin and subsequently assayed with western analysis with an anti-RhoA Ab (lower boxes). The upper blot shows total RhoA content in the input. RhoA-GTP densitometry values were adjusted to total RhoA intensity, then normalized to expression from the control sample. ** = P<0.01 vs. control. # = P<0.01 vs. E2. (B) Cells were treated with 10^−8^ M E2 for 15 min in the presence or absence of ICI 182,780 (ICI - 1 µM), of wortmannin (WM - 30 nM) or of PP2 (10 µM). ROCK-2 was immunoprecipitated with a specific Ab and the IPs were used to phosphorylate the bait protein, myelin basic protein (MBP). ROCK-2 kinase activity is shown as the amount of phosphorylated MBP (P-MBP). P-MBP densitometry values were adjusted to ROCK-2 intensity, then normalized to expression from the control sample. ** = P<0.01 vs. control. # = P<0.01 vs. E2. (C) T47-D cells were either mock-transfected or exposed to dominant-negative RhoA or constitutively active (RhoA DN or RhoA CA). Cells were then treated with E2 (10 nM) for 15 min and wild type or phosphorylated ezrin were analyzed. P-ezrin densitometry values were adjusted to actin intensity, then normalized to expression from the corresponding control sample. ** = P<0.01 vs. corresponding control without transfection. (D) Cells were exposed to 10^−8^ M E2 for 15 min after transfection with 100 nM siRNAs towards ROCK-2 or control scrambled siRNAs for 48 h. P-ezrin densitometry values were adjusted to ezrin intensity, then normalized to expression from the corresponding control sample. ** = P<0.01 vs. corresponding scrambled control. (E) Cells were exposed to 10^−8^ M E2 for 15 min after transfection with c-Src siRNAs for 48 h. Active, GTP-bound RhoA and ROCK-2 activity were assayed RhoA-GTP and P-MBP densitometry values were adjusted to total RhoA and ROCK-2 intensities respectively, then normalized to expression from the control sample. ** = P<0.01 vs. control. All these experiments were performed in triplicates and representative images are shown.

The small GTPase RhoA and its downstream effector ROCK-2 play a central role in regulating the actin cytoskeleton by the crosstalk to actin-regulatory proteins such as moesin or ezrin [6], [23]. Treatment of T47-D cells with E2 (10^−8^ M) increased the amount of active, GTP-bound RhoA (Fig. 4A) and of functionally activated ROCK-2, which was indicated by the enhanced Thr-phosphorylation of the bait protein myelin basic protein (MBP) by ROCK-2 immunoprecipitates (IPs) (Fig. 4B).

Transfection of a dominant negative RhoA construct (RhoA T19N-RhoA DN) led to a significant reduction of E2-induced ezrin phosphorylation (Fig. 4C). In constrast, ezrin phosphorylation was ligand-independently induced by transient transfection of a RhoA constitutively-active construct (RhoA G14V-RhoA CA) (Fig. 4C). Meanwhile, silencing of ROCK-2 with siRNAs prevented the E2-dependent ezrin activation (Fig. 4D).

The activation of RhoA/ROCK-2 is mediated by c-Src and PI3K, since inhibitors of both kinases blocked the activation of RhoA and ROCK-2 by E2 (Fig. 4A, 4B). In line with this, c-Src silencing with siRNAs blocked E2-induced RhoA and ROCK-2 activation(Fig. 4E), which was also impaired by transfection with Δp85α (Fig. 4F). In contrast, RhoA activation was ligand-independently increased after transfection of WT p85α (Fig. 4F).

### Intracellular events linking activation of ER to cell migration and invasion

**Figure 5:**
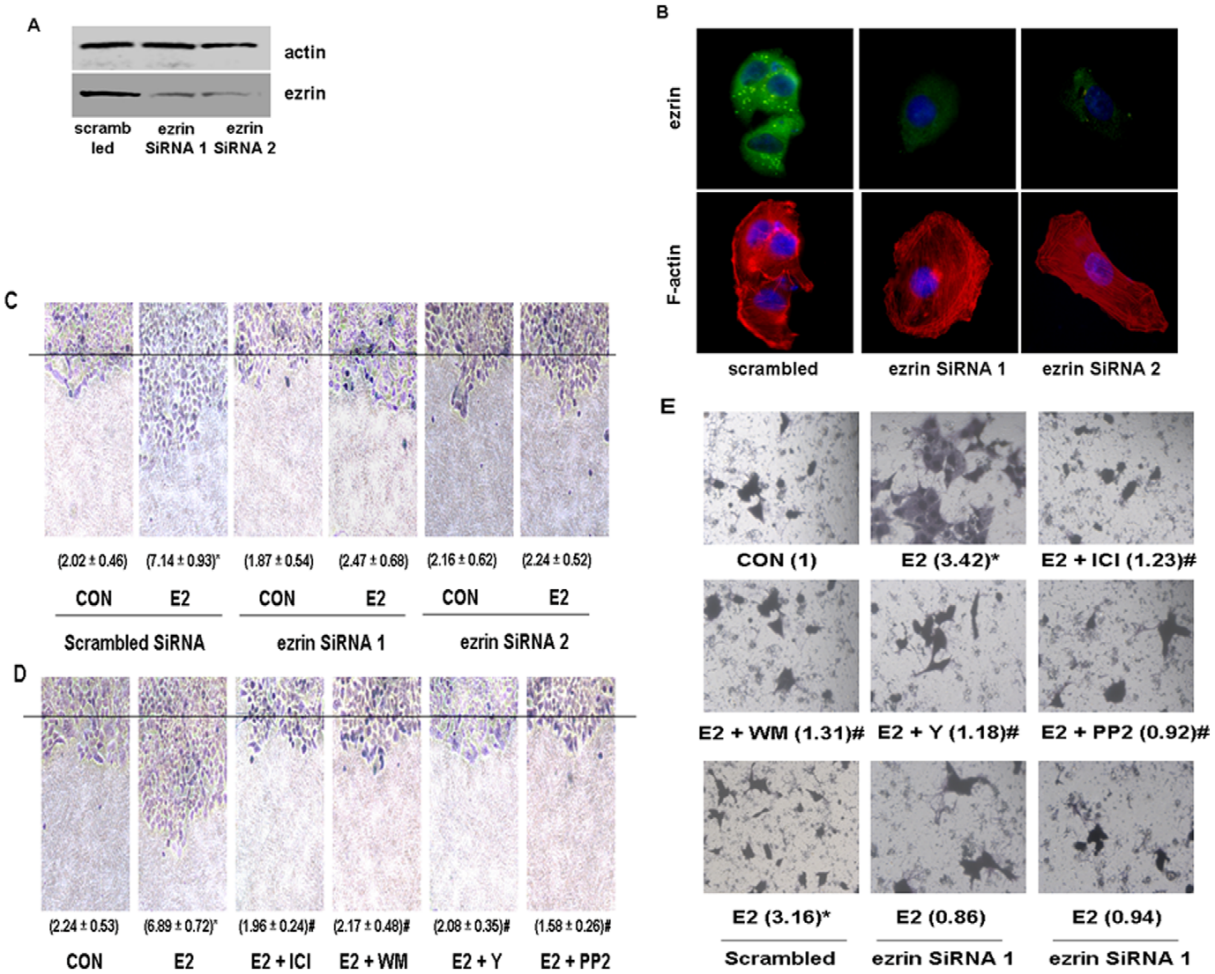
Estrogen signaling to ezrin increases T47-D cell migration and invasion. (A) T47-D cells were transfected with scrambled siRNA or ezrin targeted siRNA 1 and 2 for 48 h. Ezrin protein expression was detected by western blot and β-actin intensity was used as the loading control as indicated. (B) Cells were stained with an Ab vs. ezrin (FITC; green staining) as well as with Texas Red-phalloidin (in red). Nuclei are counterstained in blue. All the experiments were repeated three times with consistent results, and a representative result is shown. (C) Cells were transfected with 100 nM target siRNAs for ezrin or scrambled siRNA for 48 h and then treated with E2 (10 nM) for 48 h. Cell migration distances were measured and values are presented within brackets as mean migration distance (mm) ± SD. The black line indicates the starting line. * = P<0.01 vs. control. (D) Cells were treated with E2 (10 nM) for 48 h, in the presence or absence of ICI 182,780 (ICI - 1 µM), of wortmannin (WM - 30 nM), of Y-27632 (Y - 10 µM) or of PP2 (10 µM). * = P<0.01 vs. control; # = P<0.01 vs. E2. The experiments were performed in triplicates and representative images are shown. (E) T47-D cells were treated with 10 nM E2 for 24 h, in the presence or absence of ICI 182,780 (ICI - 1 µM), of wortmannin (WM - 30 nM), of Y-27632 (Y - 10 µM), of PP2 (10 µM) or after transfection with 100 nM siRNAs toward ezrin or control scrambled siRNAs for 48 h. Cell invasion was assayed using invasion chambers. Invading cells were counted in three different central fields of triplicate membranes. Statistics for invasion indexes and representative images are shown. * = P<0.01 vs. control, # = P<0.01 vs E2 without transfection. The experiments were performed in triplicates and representative images are shown.

Finally we observed the role of ezrin activation in estrogen-sensitive breast cancer cell migration and invasion. To this aim, we silenced ezrin expression by using 2 different ezrin siRNAs. Indeed, ezrin expression was largely repressed by the transfection of both siRNAs, as indicated in Fig. 5A. Furthermore, we detected the cellular ezrin expression by using immunofluoresence and it showed that the immunostaining was extremely weak in specific ezrin siRNA transfected cells, in association with a very regular arrangement of actin fibers (Fig. 5B). Consistent with our previous work [7], E2 markedly enhanced horizontal migration (Fig. 5C). This effect was completely blocked by silencing ezrin with siRNAs (Fig. 5C). The enhancement of cancer cell migration induced by E2 was also prevented by blocking ER with ICI 182,780, by blocking c-Src with PP2, PI3K with wortmannin or ROCK-2 with Y-27632 (Fig. 5D). E2 promoted breast cancer cell invasion of a three-dimensional matrix (Fig. 5E). The invasive behavior induced by E2 was prevented by silencing ezrin with siRNAs, and by the inhibitors of ER, c-Src and PI3K cascades (Fig. 5E).

## Discussion

Estrogen stimulates cell movement in different tissues, including the mammary gland. It also promotes ER^+^ breast cancer cell to invade and metastasize to lymph nodes and distant organs [24]. The key finding of the present manuscript is that estrogen signals to ezrin and induces its phosphorylation, which contributes to the enhanced ability of ER^+^ breast cancer cells to migrate and invade the surrounding environment. In parallel, it has been reported that ezrin phosphorylation is required for androgen-induced prostate cancer cell invasion [19]. Taken together, these findings help to understand the basis through which sex steroids drives gender-related cancers metastasis and may herald profound biological and medical implications.

Cell movement is a highly integrated process implemented by actin reorganization [25]. Rearrangement of actin fibers is crucial to the formation of membrane protrusions at the leading edge. This process is modulated by sex steroids in human cells, including endothelial and breast cancer cells where estrogen significantly increases the number of membrane lamellipodia and filopodia. These events are linked to the rapid activation of actin-binding protein moesin, which belongs to the ezrin/radixin/moesin (ERM) family [6], [7], [8], [9]. Notwithstanding, evidence on the link between moesin and clinical feature of cancer is limited, while more clinical trials focus on the role of ezrin in different types of cancer progression, including in breast cancer [26], [27], [28]. For example, clinical data have indicated the positive correlation between ezrin expression/abnormal localization and advancing histological grade/poor outcome [12], [13]. Moreover, the overexpression of wild-type ezrin is shown to promote breast cancer metastasis in animal model experiment [14]. Overall, these findings highlight the relevance of the ezrin activity for breast cancer progression. The identification of ezrin regulation may thus offer important mechanistic insights to breast cancer metastasis.

We here find that E2 rapidly activates Thr^567^ and Tyr^353^ phosphorylation of ezrin, leading to the actin cytoskeleton remodelling and the formation of lamellipodia and filopodia. In quiescent conditions ezrin exists in an auto-inhibited conformation. Unfolding of the molecule into an active conformation occurs following binding to phosphoinositides and phosphorylation on the carboxyl-terminal Thr^567^ [15]. The active ezrin then translocates to the plasma membrane and functions as a molecular bridge by binding membrane associated adhesion molecules and polymerized F-actin [29], resulting in actin cytoskeleton remodelling. This is confirmed by our immunofluorescent results showing that phosphorylated ezrin clusters in cell membrane lamillipodia and filopodia. It has been reported that ezrin silencing by small hairpin RNA or the expression of dominant-negative amino-terminal ezrin reverses metastatic behaviors of human breast cancer cells [14], [30], [31]. In agreement, in the present study the silencing of ezrin by small interference RNA largely impairs E2-dependent cell migration and invasion. The active ezrin is also capable to recruit the guanine nucleotide exchange factor Dbl to lipid rafts and preferentially activates Cdc42, which is important for directional breast cancer cell migration [32].

The activation of ezrin induced by E2 is achieved via extra-nuclear signaling cascades. Such mechanisms of actions depend on the recruitment of kinase cascades via the interaction of membrane ERs with specific domains of kinases, such as c-Src or PI3K [33]. Indeed, the existence of membrane-localized ERs in different tissues is well documented [34]. Although the definition of the exact intracellular E2-binding site is beyond the scope of this manuscript, there is a chance that a membrane-initiated process might be activated in this setting. In general, membrane receptor-initiated nongenomic effects have several characteristics, including the rapid time course and insensitive to mRNA and protein synthesis inhibitors [35]. Consistently, ezrin activation is rapid, which could be found after exposure to E2 for 5 minutes and this action is not blocked by Act D and CHX. Furthermore, the phosphorylation of ezrin is biphasic and this pattern of kinetics is similar to other nongenomic actions of sex steroids, such as the biphasic release of calcium [36]. The reasons for the biphasic action may be due to the rapid accumulation of second messengers when sex steroids bind to their membrane receptors. Indeed, in the present study, c-Src phosphorylation is rapidly enhanced after 2 minutes of exposure to E2 and then it gradually declines, which could explain the time-dependent attenuation of ezrin phosphorylation.

Notwithstanding, we do not exclude the longterm effects of E2 on cell migration and invasion, since it has been reported that ezrin expression is induced by E2 in other tissues [37], [38]. This makes it difficult to evaluate the overall contribution of an extra-nuclear action (such as the rapid ezrin activation) to the relevant physiologic consequences (such as the cell movement), since the blockade of the nuclear signalings, gene transcription and protein translation will negatively impact cell viability. In this regard, a recent study provided the first *in vivo* evidence that extra-nuclear ER actions prevent neointima formation in mice model by using the estrogen-dendrimer conjugate (EDC), a novel selective ER modulator (SERM) capable of exclusive non-nuclear action [39]. Therefore, the new generation of SERM, such as EDC, should be used in the future studies to address the role of extra-nuclear ER actions in hormone-sensitive breast cancer cell invasiveness.

Several signalling molecules, such as phosphatidylinositol 4,5 bis-phosphate (PIP2) and protein kinase C (PKC), have been implicated in regulating ezrin protein activity [40], [41]. The present work indicates that the non-receptor tyrosine kinase c-Src acts as a central hub in relaying ER signalling to ezrin activation. Indeed, c-Src plays important role in breast cancer cell proliferation, survival, invasion, and metastasis [42] and its activity is modulated by estrogen through multiple mechanisms. For example, c-Src is recruited and activated by the interaction of membrane ER with several scaffold proteins, such as MNAR/PELP1 and striatin [43]. Recently it has been shown that estrogen activates c-Src through the release of nitric oxide (NO), leading to breast cancer cell invasion and metastasis [44].

PI3K is a well characterized downstream effector of c-Src [45]. In our experimental setting, we identify that PI3K is the downstream of c-Src, and this step is necessary for ezrin activation, based on our findings showing that the transfection of Δp85α led to a significant reduction of E2-induced ezrin phosphorylation, while which was enhanced by transient transfection of WT p85α. Moreover, transfection with WT p85α induced a ligand-independent increase of ezrin phoshorylation, since the enhanced expression of this regulatory subunit leads to an increase of basal phoshorylated Akt [46].

This is consistent with clinical data indicating that the level of phosphorylated ezrin or the membranous ezrin staining is positively associated with the phosphorylated Akt overexpression [13], [47]. Moreover, Akt is believed to govern breast cancer metastasis, which is partly due to its modulation on ezrin phosphorylation and localization [48]. Interestingly, c-Src/PI3K can also function as the downstream cascade of ezrin to maintain cell survival or to promote breast cancer metastasis [14], [49]. Therefore, a positive feedback loop might exist between c-Src/PI3K and ezrin in breast cancer.

ER-dependent PI3K activation results in the activation of the RhoA/ROCK-2 cascade and through this manner it increases ezrin activity in breast cancer cells. A similar role of the RhoA/ROCK cascade has been established in fibroblast and in other tumor cells, where it mediates ezrin activation or redistribution [23], [50], [51]. However, the precise mechanism by which ROCK phosphorylates ezrin is not clear in this study. To this extent, there is evidence that ROCK binds and phosphorylates ERM directly [52], while other report shows that ROCK is not responsible for threonine phosphorylation of ezrin [53], [54].

Phosphorylation of ezrin is required for both conformational activation and for signaling to downstream events. Although RhoA/ROCK mediates some of the signal transduction between c-Src/PI3K to ezrin Thr^567^ phosphorylation, it may also be possible that c-Src directly interacts with ezrin and results in the phosphorylation of other residues in the presence of E2, like the Tyr^353^ phosphorylation indicated in the present study. In fact, c-Src is reported to directly phosphorylate ezrin at different sites, such as Tyr^145^ [55], Tyr^353^ [19] and Tyr^477^ [56]. For instance, it has been shown that in cells lacking Src, ezrin is not tyrosine-phosphorylated but the introduction of active c-Src restores its phosphorylation [56]. In addition, activation of c-Src results in ezrin phosphorylation and the Src tyrosine kinase inhibitor PP2 blocks this effect [57]. Although the phosphorylation of these tyrosine sites exerts different functions, recent data indicated that Tyr^353^ is an important site required for ezrin full activation of ezrin [19]. It showed that sex steroid androgen indirectly phosphorylates ezrin at Thr^567^ via PKC pathway and directly phosphorylates Tyr^353^ via c-Src, leading to ezrin full activation and promoting prostate cancer cell invasion [19]. Therefore, it is possible that E2 indirectly phosphorylates ezrin Thr^567^ via c-Src/PI3K/Akt/RhoA/ROCK-2 cascade and phosphorylates ezrin Tyr^353^ via a direct interaction of c-Src and ezrin. The full characterization of the functional interrelation between c-Src and ezrin during estrogen exposure is therefore not solved and will require future studies.

As we mentioned before, the mechanisms that estrogen affects breast cancer metastasis are complex, including nongenomic and genomic actions. Through nongenomic activation of actin-binding proteins, including moesin [7] and focal adhesion kinase [58], estrogen rapidly provoke actin cytoskeleton reorganization in breast cancer cells, leading to the formation of membrane specialized structures that facilitate breast cancer cell migration and invasion, as we indicated in this work. Meanwhile, estrogen transactivate receptor tyrosine kinases (including epidermal growth factor receptor and insulin-like growth factor receptor), resulting in growth factor-like effects that transform cancer cell into an invasive phenotype [59]. Moreover, the expression patterns of metastasis-associated molecules, such as cell adhesion molecule E-cadherin [60], matrix metalloproteinases [61], growth factors [62], [63], chemokines and their receptors [64], [65], are reported to be regulated by estrogen, leading to epithelial-to-mesenchymal-like transition and promoting breast cancer cells escape from primary site and migrate toward target tissues and organs. In this study, we show that E2 promotes breast cancer cell movement and invasion by the rapid activation of ezrin. In the presence of E2, ER activates c-Src and PI3K/Akt, leading to RhoA/ROCK-2 activation, which eventually phosphorylates ezrin at Thr^567^. Our results provide original mechanistic insights into the effects of E2 on breast cancer progression and may in the future be helpful to develop new drugs against endocrine-sensitive breast cancers.

## Materials and Methods

### Cell cultures and treatments

T47-D breast cancer cells were obtained from China Center for Type Culture Collection (www.cctcc.org) and incubated in phenol red-free RPMI 1640 medium containing 10% fetal calf serum (FCS), 0.2 UI/mL insulin, L-glutamine and penicillin streptomycin under a 5% CO_2_ atmosphere at 37°C. Before experiments investigating non-transcriptional effects, cells were kept in phenol red-free DMEM containing no FBS for 8 hours. Whenever an inhibitor was used, the compound was added 30 minutes before starting the treatments. 17β-estradiol (E2), pertussis toxin (PTX), Y-27632, PD98059 and wortmannin were from Sigma-Aldrich (Saint-Louis, MO). 4-amino-5-(4-chlorophenyl)-7-(t-butyl) pyrazolo (3,4-d) pyrimidine (PP2) was from Calbiochem (EMD Biosciences, Germany). 4′,4″,4′″-(4-propyl-[1H]-pyrazole-1,3,5-triyl) trisphenol (PPT), 2,3-bis-(4–ydroxyphenyl)-propionitrile (DPN) and ICI 182 780 were purchased from Tocris Cookson (Bristol, UK).

### Immunoblottings

Cell lysates were separated by SDS-PAGE. Antibodies used were: phospho-Thr^567^-Ezrin (A00334, Genescript, Aachen, Germany), ezrin (#3145 ,Cell signaling), phospho- Tyr^416^-Src (# 2101, Cell Signalling Technology), Src (# 2108, Cell signaling) , ROCK-2 (C-20, Santa Cruz Biotechnology, Santa Cruz, CA, USA), phosphor- Ser^473^- Akt (Upstate Biotechnology, Inc., Lake Placid, NY), actin (sc-1615, Santa Cruz), Akt (# 9272, Cell Signalling Technology). Primary and secondary Abs were incubated with the membranes with standard technique. Immunodetection was accomplished using enhanced chemiluminescence. Chemiluminescence was acquired with a quantitative digital imaging system (Quantity One, BioRad, Hercules, CA) allowing to check for saturation. Overall emitted photons were quantified for each band, particularly for loading controls, which were homogeneously loaded.

### Kinase assays

T47-D cells were harvested in 20 mM Tris-HCl, 10 mM EDTA, 100 mM NaCl, 0.5% IGEPAL and 0.1 mg/mL PMSF. Equal amounts of cell lysates were immunoprecipitated with Rhotekin RBD agarose (Rhotekin is the protein that binds specifically to GTP-bound, and not GDP-bound, RhoA. The Rhotekin-RBD contains residues 7–89 of Rhotekin. This region includes the sequences required for the high affinity interaction with GTP-Rho. # 14-383, upstate) *vs*. GTP-RhoA or an Ab *vs*. ROCK-2 (C-20, Santa Cruz). The IPs were washed three times with buffer containing 20 mM Tris-HCl, 10 mM EDTA, 150 mM NaCl, 0.1% IGEPAL and 0.1 mg/mL PMSF. For ROCK-2 activity assay, two additional washes were performed in kinase assay buffer (20 mM MOPS, 25 mM β-glycerophosphate, 5 mM EGTA, 1 mM DTT) and the samples were therefore resuspended in this buffer. 5 µg of de-phosphorylated myelin basic protein (Upstate) together with 500 µM ATP and 75 mM MgCl_2_ were added to each sample and the reaction was started at 30°C for 20 min. The reaction was stopped on ice and by resuspending the samples in Laemmli Buffer. The samples were separated with SDS-PAGE and Western analysis was performed using antibodies recognizing RhoA (sc-418, Santa Cruz) or Thr^98^-P-myelin basic protein (05-429, Upstate).

### Cell immunofluorescence

T47-D breast cancer cells were grown on coverslips and exposed to treatments. Cells were fixed with 4% paraformaldehyde for 30 min and permeabilized with 0.1% Triton X for 5 min. Blocking was performed with 3% normal serum for 20 min. Cells were incubated with antibody against phospho-Thr^567^-Ezrin (A00334, Genescript) and FITC-conjugated secondary antibody (K00018968, Dako North America Inc., Dako, Denmark). After washing the nuclei were counterstained with 4′-6-diamidino-2-phenylindole (DAPI) (Sigma) and actin was stained with Texas Red-phalloidin (Sigma). The coverslips were mounted with Vectashield mounting medium (Vector Laboratories, Burlingame, CA). Immunofluorescence was visualized using an Olympus BX41 microscope and recorded with a high-resolution DP70 Olympus digital camera. Pictures were photographed.

### Transfection experiments

On-TARGETplus SMARTpool siRNA reagents against human Src (NM-198291), ROCK-2 (NM-004850), ezrin (NM-017370, siRNA 1) and control siRNA (D-001810-01-05) were purchased from Dharmacon (Thermo Fisher Scientific Inc, USA). In order to eliminate the off-target effect, another ezrin siRNA (sc-35349, siRNA 2) purchased from Santa Cruz was also used. T47-D cells were transfected with siRNA using Lipofectamine (Invitrogen) according to the protocol. Cells (40% confluent) were serum-starved for 1 h followed by incubation with 100 nM target siRNA or control siRNA for 6 h in serum-free media. The serum-containing media was then added (10% serum final concentration) for 42 h before experiments and/or functional assays were conducted. Target protein silencing was assessed through protein analysis up to 48 h after transfection.

Each plasmid (15 µg) was transfected into T47-D breast cancer cells using the Lipofectamine (Invitrogen) according to the manufacturer's instructions. The transfected plasmids were as follows: RhoA T19 and RhoA G14V, p85α or dominant-negative p85α (Δ p85α). These constructs were obtained from the Guthrie cDNA Resource Center (www.cdna.org). All the inserts were cloned in pcDNA3.1+. As control, parallel cells were transfected with empty pcDNA3.1+ plasmid. Cells (60–70% confluent) were treated 24 h after transfection, and cellular extracts were prepared according to the experiments to be performed.

### Cell migration assays

Cell migration was assayed with razor scrape assays as previously described [9]. Briefly, a razor blade was pressed through the confluent T47-D breast cancer cell monolayer into the plastic plate to mark the starting line. T47-D cells were swept away on one side of that line. Cells were washed, and 2.0 mL of DMEM containing steroid-deprived FBS and gelatin (1 mg/mL) were added. Cytosine β-D-arabinofuranoside hydrochloride (Ara-C, Sigma) (10 µM), a selective inhibitor of DNA synthesis which does not inhibit RNA synthesis was used 1 h before the test substance was added. Ara-C is easy to enter into the cellular nucleus and it incorporates into DNA and inhibits DNA replication by the formation of cleavage complexes with topoisomerase I resulting in DNA fragmentation. Although the half-life of Ara-C was less than 1 h in most of the cell lines, more than 80% of Ara-C DNA was retained at 24 h after drug removal [66]. Migration was monitored for 48 hours. Every 12 h fresh medium and treatment were replaced. Cells were digitally imaged and migration distance (mm) was measured by using phase-contrast microscopy.

### Cell invasion assays

Cell invasion were assayed following the standard method by using the BD BioCoatTM Growth Factor Reduced (GFR) Matrigel™ Invasion Chamber (BD Bioscience, USA). In brief, after rehydrating the GFR Matrigel inserts, the test substance was added to the wells. An equal number of Control Inserts (no GFR Matrigel coating) were prepared as control. 0.5 mL of T47-D cell suspension (2.5×10^4^ cells/mL) were added to the inside of the inserts. The chambers were incubated for 24 h at 37°C, 5% CO_2_ atmosphere. After incubation, non-invading cells were removed from the upper surface of the membrane using cotton tipped swabs. Then the cells on the lower surface of the membrane were stained with Diff-Quick stain. The invading cells were observed and photographed under the microscope at 100× magnification. Cells were counted in the central field of triplicate membranes. The invasion index was calculated as the % invasion test cell/% invasion control cell.

### Statistical analysis

All values are expressed as mean ± SD. Statistical differences between mean values were determined by ANOVA, followed by the Fisher's protected least significance difference (PLSD). All differences were considered significant at P<0.05.
